# The Usefulness of Pine Timber (*Pinus sylvestris* L.) for the Production of Structural Elements. Part II: Strength Properties of Glued Laminated Timber

**DOI:** 10.3390/ma13184029

**Published:** 2020-09-11

**Authors:** Radosław Mirski, Dorota Dziurka, Monika Chuda-Kowalska, Jakub Kawalerczyk, Marcin Kuliński, Karol Łabęda

**Affiliations:** 1 Department of Wood Based Materials, Faculty of Wood Technology, Poznań University of Life Sciences, Wojska Polskiego 38/42, 60-627 Poznań, Poland; jakub.kawalerczyk@up.poznan.pl (J.K.); kmarcin97@gmail.com (M.K.); 2 Institute of Structural Analysis, Faculty of Civil and Transport Engineering, Poznan University of Technology, pl. Sklodowskiej-Curie 5, 60-965 Poznań, Poland; monika.chuda-kowalska@put.poznan.pl; 3 Department of Furniture, Faculty of Wood Technology, Poznań University of Life Sciences, Wojska Polskiego 38/42, 60-627 Poznań, Poland; karol.labeda@up.poznan.pl

**Keywords:** beams, glued laminated timber, modulus of elasticity, pine wood, laboratory tests

## Abstract

The paper assessed the feasibility of manufacturing glued structural elements made of pine wood after grading it mechanically in a horizontal arrangement. It was assumed that the pine wood was not free of defects and that the outer lamellas would also be visually inspected. This would result in only rejecting items with large, rotten knots. Beams of the assumed grades GL32c, GL28c and GL24c were made of the examined pine wood. Our study indicated that the expected modulus of elasticity in bending was largely maintained by the designed beam models but that their strength was connected with the quality of the respective lamellas, rather than with their modulus of elasticity. On average, the bending strength of the beams was 44.6 MPa. The cause of their destruction was the individual technical quality of a given item of timber, which was loosely related to its modulus of elasticity, assessed in a bending test. Although the modulus of elasticity of the manufactured beam types differed quite significantly (11.45–14.08 kN/mm^2^), the bending strength for all types was similar. Significant differences occurred only during a more detailed analysis because lower classes were characterized by a greater variation of the bending strength. In this case, beams with a strength of 24 MPa to 50 MPa appeared.

## 1. Introduction

Developments in the construction industry and searching for ways to use conventional and alternative structural materials have provided new materials: EWPs (Engineering Wood Products). In the case of EWPs, the idea is to obtain a full-quality product from a material that was originally not suitable for specific uses due to its size or insufficient quality [1,2]. Nowadays, Europe and the world have seen developments in the technology of the manufacturing and application of glued timber, mainly GLT (Glued Laminated Timber). This material fits very well with the EWP technology trend. GLT has the typical features of solid timber: light weight, good strength, elasticity, durability, easy processing and a unique feature, i.e., it is readily shaped into cross-sections. Its cross-section has a layered structure, enabling the manufacturing of components with variable cross-sectional heights, as needed [3,4,5,6].

Wooden components are glued with binding agents that guarantee a high strength under static and dynamic loads. Structural timber layers, especially those for making load-bearing structures, are nearly always combined by means of resorcinol-phenol-formaldehyde (PRF) or melamine-urea-formaldehyde (MUF) glues. Apart from these, polyurethane-based glue is becoming more and more popular. All these adhesives should provide a high durability in variable environmental conditions [7,8,9,10,11].

Studies on the durability of straight and bent beams composed of wooden layers glued together with synthetic resins indicate that such elements have comparable load-bearing capacities to those made of solid timber. Moreover, the layered timber structure has resulted in an improved quality of the material [3,12,13,14,15,16,17,18,19,20,21].

Recent research studies on glued laminated timber have focused, among other things, on materials glued lengthwise and crosswise. Test results for high-grade solid timber and glued laminated timber, even that obtained from lower-quality timber after grading, indicate that the latter has better strength properties. This is mostly attributed to the distribution of defects and to the gluing process. For instance, the studies focused on the shaping of cross-sections of items made out of thin timber that was comprised of different grade materials with respect to their strength [22,23,24,25,26,27,28,29,30].

According to JCR (Journal Citation Reports), more than 700 scientific papers on glulam timber were published in the last 20 years. They considered, among other things, the component’s reinforcement methods by using glass or carbon fibers [31,32,33,34], the effect of different factors on the behavior of steel-timber composites (STC) [35,36,37] and glulam-concrete beams [38,39,40].

As mentioned earlier, in the case of glued laminated timber, the cross-sections of the obtained elements can be shaped as required. However, what is important is that the strength is also improved, and it is generally higher than the combined elements. The coefficient of variation for the bending test is also improved [41,42]. In the works of Tomasi [43] and Gonzales [37], a significant improvement in the mechanical properties of glued laminated beams reinforced with steel rods was indicated. However, they pointed out that the quality of the steel-wood joint was of significant importance in these systems. The abovementioned authors draw attention to systems made of different lamella qualities; however, there is the possibility of having the lamellas be joined in their width [44,45] or having them made of different species [46,47]. The created systems result from certain fixed concepts, as described in appropriate standard or the model described in the works of Fink [48], Foschi and Barrett [49], or Hernandez et al. [50] may be the basis for their creation. Predicting the future quality of the produced GLT elements is an overriding task for engineers who support this branch of the wood industry because, in contrast to the mass production of wood-based panels with stabilized parameters, testing the produced GLT is expensive and difficult.

**Figure 1:**
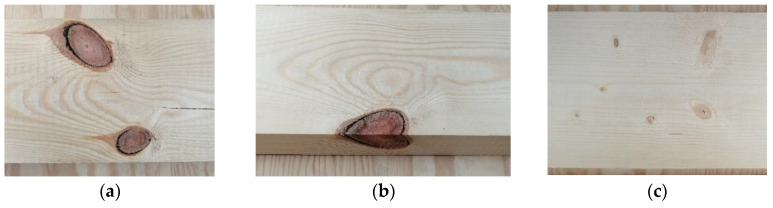
The examples of knots from: (**a**,**b**) Scots pine and (**c**) Norway spruce.

The parameter of GLT which determines its suitability is, first of all, the strength of lamellas with different parameters and properties, as defined in applicable normative documents, taking into consideration the acceptable classes of glued timber [51,52,53]. The current strength classification system (EN 338) [54] for structural timber enables the use of a single-strength class in the range of C16–C30 for beams of uniform structure and in combination with lower classes C16 and C18 in the case of a nonuniform structure (EN 384) [55]. The class C is related to the static bending strength of manufactured material. The strength of defect-free pine wood is usually in the range of 90–110 MPa. However, because of its natural features, which from a technological perspective are often recognized as defects, the mechanical properties deteriorate considerably. The strength characteristics of the majority of timbers are often below 20 MPa, which results mostly from the occurrence of knots. They appear in various sections with an interval of 40 to 60 cm. The timber properties are affected not only by the number and size of the knots, but also by their soundness. Low-quality knots are usually cut, and the obtained elements are combined with the use of finger joints. This technique is known and has been constantly developed since the Second World War [56]. The knots are an important type of defect and due to their dimensions affect in particular the pine wood (Figure 1a,b). For comparison, a spruce timber is characterized by the presence of significantly smaller knots (Figure 1c). Thus, their removal contributes to an improvement in both the technical quality and visual aspects of each piece of pine timber. However, it seems like, in the case of glued components, the occurrence of knots has a smaller effect and the visual side is less important.

Consequently, the aim of the presented work was to investigate the possibility of using pine timber sorted solely on the basis of mechanical properties, with the exception of the outer layers. The outer lamellas of the eight-layer beams were also assessed visually. During the assessments, the pieces of timber having edge knots or large rotten knots were classified as unsuitable for the outer layers.

## 2. Materials and Methods

The research material was pine wood with the following dimensions: 137 mm wide × 39.50 mm thick × 3485 mm long. The average density of the timber items was 571 kg/m^3^ (average moisture 8.98%). The pine wood was obtained by sawing timber in the form of logs having round cross sections and originating from the Forest Division Olesno (50°52′30″ N 18°25′00″ E). The obtained sawn timber was dried to a moisture content of 10% ± 2%. After drying, the sawn timber was organized so as to obtain a uniform thickness of all the lamellas. The preliminary assessment was performed in accordance with EN 338. The detailed description of the modulus of elasticity assessments is included in the first part of the research. Selected timber items were used for the preparation, in semi-industrial conditions, of glued beams with a diameter of 137 mm × 300 mm, i.e., comprising eight layers. With the exception of the outer layers, the choice of the lamellas for making the beams depended only on the determined value of the modulus of elasticity. The outer layers, with the exception of the required value of the modulus of elasticity, were required to have no edge knots. The raw material originating from that region is characterized by a higher percentage of timber, whose physico-mechanical parameters enable a considerable portion of it (45%) to be classified into higher classes than C24 (details will follow in the next chapter). Therefore, it was assumed that the respective beam models would satisfy the conditions for a modulus of elasticity set out for grades GL24c, GL28c and GL32c according to EN 14,080 [57]. The elastic properties of the beams layers were determined according to Bodig and Jayne [58], assuming that the beam was symmetric and contained eight lamellas (1):

(1) $$E_{ef}=\frac{1}{J_{y}}\sum_{i=1}^{4}E_{i}\left[J_{yi}+A_{i}{\left(d_{i}\right)}^{2}\right]$$

where:

*E_ef_*—effective/substitute modulus of elasticity, N/mm^2^,
*J_y_*—area moment of inertia, mm^4^,
*E_i_*—modulus of elasticity of layer, N/mm^2^,
*A_i_*—cross-sectional area, mm^2^,
*d*—distance from the neutral axis, mm.

The adopted values of the modulus of elasticity for various types of beams are shown in Table 1.

Just before being used for the preparation of glued beams, the timber items were further processed via a plan to improve their surfaces before gluing them together. The effective thickness of individual lamellas was 37.5 mm. The resulting surface was covered with an amount of glue of 220–250 g/m^2^. Melamine-urea-formaldehyde resin (MUF 1247) and its dedicated hardener (2526), both from Akzo Nobel (Amsterdam, Netherlands), were used as the binding agent. The mixture was prepared while taking into account the conditions prevailing in the laboratory room. The hardener was used at 20 g per each 100 g of resin, as recommended by Akzo Nobel for that resin. The glue was applied using a roller coating machine. The beams were manufactured at a room temperature range between 20 °C and 24 °C. The press loading time was around 12–15 min. Four beams were pressed at the same time under a pressure of 0.48 MPa for 20 h. Four beams were manufactured each day. Pressing was conducted with the use of an industrial press equipped with hydraulic cylinders dedicated to the production of glued, structural elements (FOST, Czersk, PL). After production, the beams were air-conditioned in the laboratory for min. four weeks. The conditions in the laboratory were controlled: the temperature was 21 ± 2 °C, and the air humidity was 55–65%. After the period of air conditioning, the beams were assessed for their mechanical properties. Due to the weight of the beams, they were not planed. Excess glue was manually removed immediately prior to testing the mechanical properties.

**Figure 2:**
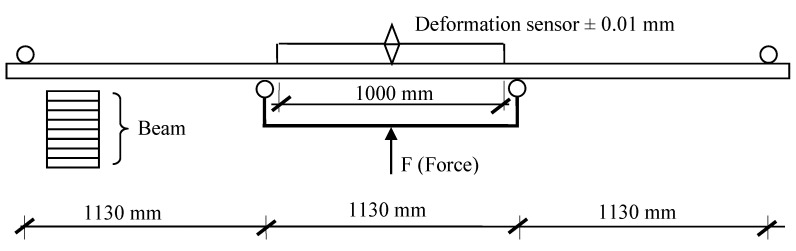
The scheme of the tested eight-layered beam.

**Figure 3:**
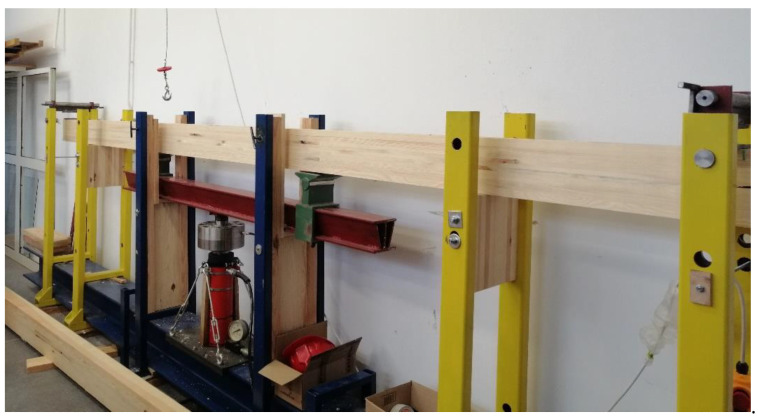
Test stand for the evaluations of the bending strength and modulus of elasticity.

The resulting beams were evaluated for their 4-point bending strength, in accordance with the diagram shown in Figure 2. Figure 3 shows the appearance of the test stand. It was equipped with: a hydraulic cylinder (50 Mg, Hi-Force, Daventry, UK), hydraulic pump (50 Mg, Hi-Force, Daventry, UK), oil flow rate regulator (Hi-Force, Daventry, UK), force sensor (CL 16 tm 500 kN, ZEPWN, Marki, PL) and deformation sensor (KTC-600-P, Variohm Eurosensor, Towcester, UK). 

In order to take into account the influence of the moisture content on the modulus of elasticity, the obtained results were calculated in accordance with Bauschinger’s Equation (2):

(2) $$E_{12}=E_{MC}\left[1+\alpha_{MC}\cdot \left(MC-12\right)\right]$$

where:

*E_12_*—modulus of elasticity of wood for a moisture content of 12%, N/mm^2^,
*E_MC_*—modulus of elasticity of wood for a moisture content of 4% < w < 20%,
*α_MC_*—coefficient of variation of the modulus of elasticity of wood after its moisture content changed by 1%—assumed to be 0.02,
*MC*—absolute moisture content of wood, %.

The destructive test included the assessment of the point and cause of failure for each specific beam.

The results of the experimental measurements were analyzed using the STATISTICA 13.0 package (Version 13.0, StatSoft Inc., Tulsa, OK, USA).

## 3. Results and Discussion

The mean values of the modulus of elasticity are shown in Table 2. The values shown therein indicate that the prepared beams, with the exception of grade GL32c, exhibited a low variability of the modulus of elasticity in bending. Moreover, the obtained values were close to or only slightly higher than the assumed ones (negative value of δ). Since the moisture of the beams during the test differed considerably from 12% (the average moisture for all the beams was 8.83%), the outcomes were recalculated using Bauschinger’s Equation (2). With the exception of grade GL32c beams, the calculated values of the modulus of elasticity were only slightly lower than the assumed ones. For GL32c, the relative difference was 5.1%. Assuming that the values of the modulus of elasticity calculated for 12% MC are appropriate, it should be expected that the beams satisfy the assumptions in this regard.

**Figure 4:**
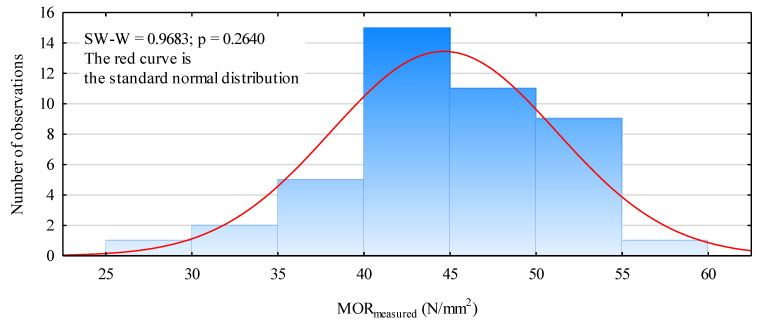
A histogram of the static bending strength for the glued beams made of mechanically graded timber.

It is assumed that the prepared beams should have a static bending strength that is not lower than 24 N/mm^2^, 28 N/mm^2^ and 32 N/mm^2^, respectively, for beam types GL24c, GL28c and GL32c. The lowest strength for all the prepared beams was 29.97 N/mm^2^, and the highest was 55.38 N/mm^2^. However, the static bending strength of the beams had a normal distribution (Figure 4), and, importantly, its standard deviation was only 6.45 N/mm^2^ and its variation coefficient was 14.5%, even though they were designed for different values of the modulus of elasticity. This means that the strength of the obtained beams was characterized by a low variability and was not strongly correlated with the designed system.

Hence, the static bending strength is not correlated with the grade of the designed beams.

**Figure 5:**
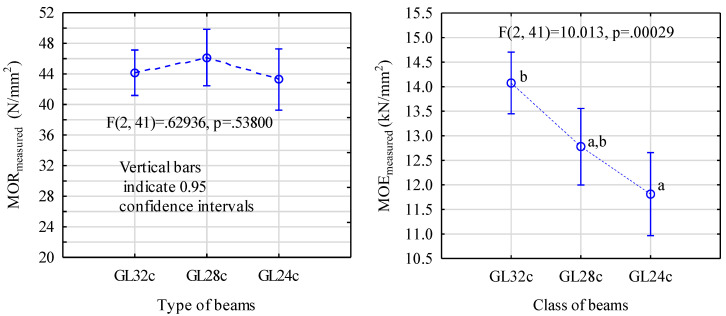
A one-factor ANOVA for the system: glued beam’s grade—static bending strength; and beam’s grade—modulus of elasticity. The letters denote uniform groups for Tukey’s test.

The data in Figure 5 show that all the models are characterized by a similar strength of around 44.5 N/mm^2^, regardless of the assumed timber grade, whereas an analysis of the modulus of elasticity shows the presence of two clearly different groups.

The values of strength obtained in the bending test were also recalculated with Bauschinger’s formula, using a factor of α = 0.04 this time. The results obtained with that factor are shown in Figure 5. The mean values calculated for all the beams were thus reduced from 44.5 N/mm^2^ to 38.6 N/mm^2^, which is still rather high. However, the assignment to the specific grade GL is based on a 5-percentile value of strength. For the represented number of samples, this value is the lowest or close to the lowest.

**Figure 6:**
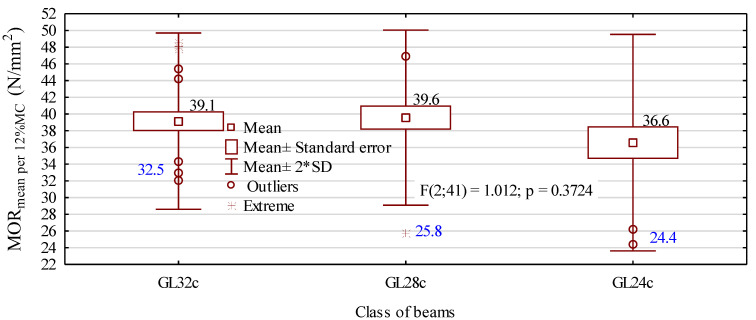
Characteristic values of the static bending strength for the prepared beam (samples). The numbers in blue denote the 5-percentile value.

The values shown in Figure 6 indicate that the beams assigned to the groups GL32c and GL24c satisfied the strength requirement, reaching the following values: 32.5 N/mm^2^ for grade GL32c beams and 24.4 N/mm^2^ for grade GL24c beams. The batch of beams modeled to be assigned to grade GL28c did not satisfy requirements and should have been assigned to grade GL24c, even though it had the highest mean value. What is important, in the case of that group, is that its assignment to the specific grade was attributed to a value regarded as being a statistical extreme. Moreover, the second lowest value of the static bending strength reached in that group was as high as 36.8 N/mm^2^. Without taking into account the strength of the three beams with the lowest values, the 5-percentile value would be 32.5 N/mm^2^.

**Figure 7:**
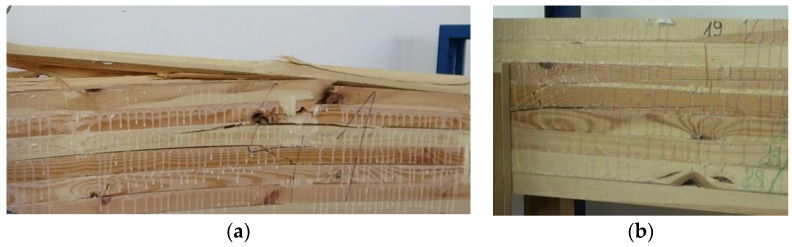
Beam failure images: (**a**) GL24c—MOR(MOR_12%_)—48.5(43.4) N/mm^2^—the strongest in the group, (**b**) GL28c—MOR(MOR_12%_)—30.4(25.8) N/mm^2^—the weakest in the group.

It is hard to predict the exact point of failure and the potential strength in some cases. For Beam 41 (Figure 7a), the cause of destruction was found, as expected, in the second and third lamellas and was due to the presence of large rotten knots in the pure bending zone. On the other hand, the beam demonstrated a strength that was nearly twice as high as expected. In the second case, failure occurred in the middle zone, for the lamellas 3/4/5 from the top, in a practically knotless zone, at a strength of about 98 kN (Figure 7b).

**Figure 8:**
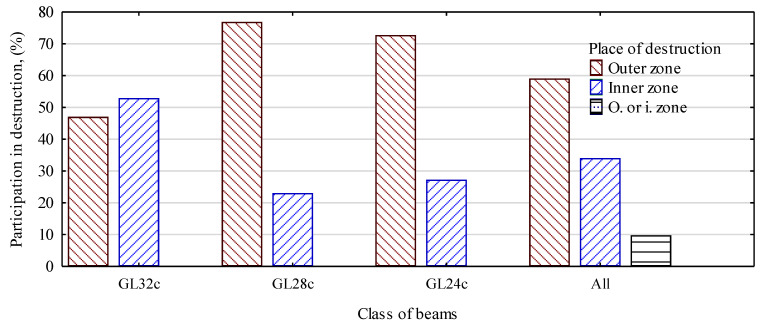
Impact of the point of destruction for various beam grades.

Obviously, the presence of knots is the main cause of the beams’ destruction. On the other hand, nearly 60% of the beams failed because of damage of the outer lamellas, and some 34% failed because of damage of the middle lamellas. For three beams, the exact starting point of destruction could not be identified (Figure 8).

**Figure 9:**
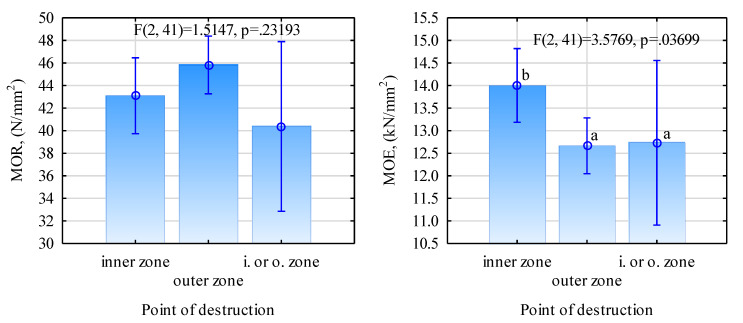
ANOVA of the assessment of the static bending strength and modulus of elasticity relative to the point of destruction (Letters mark uniform groups determined with the Tukey HSD test).

The type of destruction propagating from the beam’s middle zone was only dominant for grade GL32c beams. In the other cases, more than 70% involved destruction in the outer layer. It would be unjustified to reject the zero hypothesis that states that the strength of the prepared beams depends on the starting point of the propagation of the destruction (Figure 9). The average static bending strength for the beams destroyed as a consequence of damage to the outer lamellas was 39.6 N/mm^2^, and the value was 37.3 N/mm^2^ for those where the destruction originated in the middle layer. A different situation was observed for the modulus of elasticity. In this case, the differences were statistically significant and beams with a higher MOE value were destroyed mainly in the middle layer. This is probably attributable to the fact that the beams with higher moduli of elasticity had higher-quality outer lamellas and were capable of withstanding the arising stress, whereas lower-grade timber, though located deeper, was exposed to critical/damaging stress.

**Figure 10:**
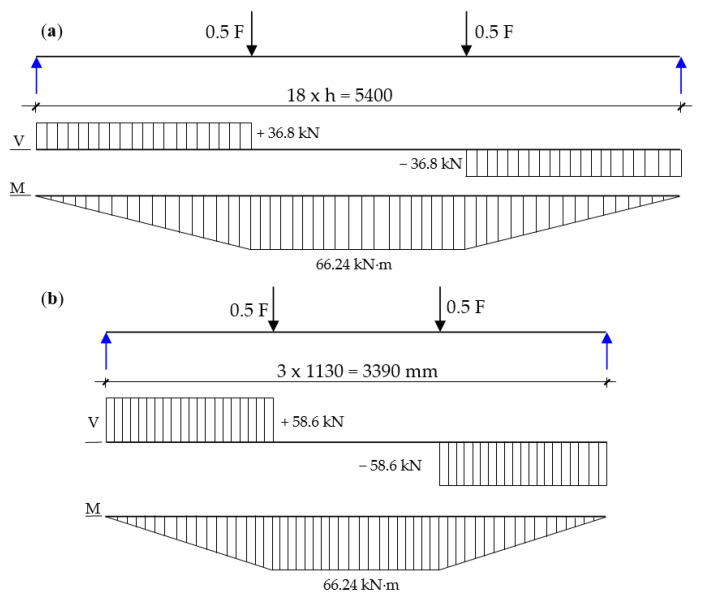
Shear forces diagrams and bending moments diagrams for (**a**) beams consistent with the dimensional requirements of EN 408 and (**b**) experimental beams used in the conducted research.

In order to determine the effect of the relation between the supports’ spacing (l) and the height of the manufactured beams (h), a model system with the cross-sections’ dimensions of 138 × 300 mm and a strength of 32 MPa was adopted. The J_y_ value for the adopted system was 31,050 cm^4^. According to EN 408 [59], the l/h relation should be 18 ± 3; however, in the conducted research, the beams were characterized by a relation of 13.3. The shear forces diagrams and bending moments diagrams of the beams, with a support spacing of 18 × h = 5400 mm (in compliance with the standard) and with a support spacing of 3390 mm for experimental beams, are presented in Figure 10. Moreover, the significant physical quantities’ characteristics for the bend test are included in Table 3.

On the basis of the results presented in Figure 10 and Table 3, it can be concluded that the significant reduction in the beams’ length in comparison with the regulations of EN 408 [59] showed an increase in the shear stresses by 60%. The considerable increase in the shear force value can lead to the beams’ destruction in the inner zone, more precisely in the inner lamellas. The assumed timber length (and consequently also the beam length) derived from the most efficient breakdown of the 14-m long logs into 3.5-m long sections. This type of division ensured less material wastes and made it easier to move the research materials; however, this length can influence the obtained results. The average shear strength of pine wood is around 10 N/mm^2^ (it ranges between 6 and 14 N/mm^2^). However, our observations show that this did not have a significant impact on the obtained research results. Most of the beams were destroyed between the pressures. In exceptional cases, the beams were damaged outside the pressures, but mainly in the tension zone.

For solid structures or homogeneous glued laminated timber, the stress distribution will be linear over the entire height of the section (GL32h—Table 4). Composite beams are constructed from more than one type of material so as to increase stiffness or strength (or to reduce cost). In the analyzed case (e.g., GL32c—Table 4), the layers are glued together. Therefore, it should be assumed that the deformations at the interface of the layers are the same. In the elastic range at the height of each layer, the stress distribution will be linear. However, due to the variable E modulus for each layer, we observe stress jumps at the layers’ borders.

It appears that stress jumps at the layers’ borders, although not large, may contribute to the destruction of the beams in the deeper lamellas. Deeper lamellas were clearly damaged where there were wood defects. In the case of very high-quality external lamellas, the second and third layers were responsible for the quality of the beam. It should be remembered that the lamellas of these layers were only assessed in terms of the linear elasticity modulus.

## 4. Conclusions

For eight-layered beams made of grade GL24c, GL28c and GL32c timber, the modulus of elasticity was only slightly different from its assumed values, and the obtained beams satisfied the requirements of the standard EN-14080 [57] in this regard.

The static bending strength obtained from the 4-point bending test was not related to the class of the designed beam models. Regardless of the assumed class, the average beam strength was above 36.6 N/mm^2^.

We adopted a procedure for preparing timber before making glued components, which provided the designed systems with satisfactory modulus of elasticity values and a static bending strength that was essentially higher than its assumed value. However, the scope of the study needs to be extended to include beams with other cross-sections so that the objective of this research work can be fully met. Nonetheless, at this point in our research, it seems that the visual grading of timber can be limited to only the timber items that are intended for use as outer layers.

## Figures and Tables

**Table 1:** Elastic properties of designed beams.

Beam Type	Number of Samples	E_mean_	E_min_	E_max_	E_mean 1st layer_	E_mean 4st layer_	MOR_declaration *_
		kN/mm^2^					N/mm^2^
GL24c	12	11.71	11.25	11.93	12.53/1.42 **	8.48/1.88	24
GL28c	14	12.82	11.98	13.50	13.96/5.44	8.08/11.69	28
GL32c	22	14.84	14.13	16.52	16.45/8.64	8.58/11.06	32

* Modulus of rupture (MOR)—characteristic value according to EN 14,080 [47], ** CoV (coefficient of variation).

**Table 2:** Elastic properties of the designed beams.

Beam Type	Assumed Values		Determined Values		δ * (%)	E_mean per 12%MC_ (kN/mm^2^)	E_5percentyl per 12%MC_ (kN/mm^2^)
	E_meanZ_ (kN/mm^2^)	CoV (%)	E_meanP_ (kN/mm^2^)	CoV (%)			
GL24c	11.71	1.81	12.79	6.42	−9.21	11.45	10.43
GL28c	12.82	3.83	13.63	6.84	−6.31	12.78	11.78
GL32c	14.84	4.00	14.94	14.1	−0.68	14.08	11.68

*—Relative change: δ = (E_meanZ_ − E_meanP_)/E_meanZ_ × 100%.

**Table 3:** Physical quantities for the bend test—No. 1 and 2: theoretical beams, No. 3: a beam with the maximum strength obtained during the tests.

No.	Type of Beam (mm)	Class of MOR (N/mm^2^)	F (N)	V (kN)	M (kN·m)	τ_xz_ (N/mm^2^)
1	18 × h = 5400	32	73,600	36.8	66.2	1.3
2	11.3 × h = 3390	32	117,240	58.6	66.2	2.1
3	11.3 × h = 3390	48.6	154,770	77.4	66.2	2.8

**Table 4:** Modules of elasticity of individual layers for beams of the Gl32h (homogeneous) and GL32c (combined) type and their stress diagrams.

**Beams**	**GL32h**			**GL32c**			
	***E*** **(N/mm^2^)**	**σ_x_ (N/mm^2^)**		*E* * **(N/mm^2^)**	**σ_x_ (N/mm^2^)**		
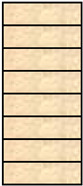		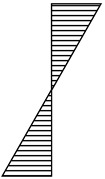	32.2		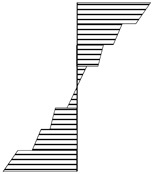		35.7
	14,200			16,450		21.6	26.8
	14,200			13,270		12.4	14.4
	14,200			11,420		4.66	6.20
	14,200		0	8580		0	
	14,200			8580			0
	14,200			11,420		6.20	4.66
	14,200			13,270		14.4	12.4
	14,200			16,450		26.8	21.6
			32.2			35.7	

*—laboratory beam GL32c.
